# A novel simulation-based analysis of a stochastic HIV model with the time delay using high order spectral collocation technique

**DOI:** 10.1038/s41598-024-57073-3

**Published:** 2024-04-04

**Authors:** Sami Ullah Khan, Saif Ullah, Shuo Li, Almetwally M. Mostafa, Muhammad Bilal Riaz, Nouf F. AlQahtani, Shewafera Wondimagegnhu Teklu

**Affiliations:** 1 Department of Mathematics, City University of Science and Information Technology, Peshawar, KP 25000 Pakistan; 2 Department of Mathematics, University of Peshawar, Peshawar, KP 25000 Pakistan; 3 School of Mathematics and Data Sciences, Changji University, Changji, Xinjiang, 831100 People’s Republic of China; 4 Department of Information Systems, College of Computers and Information Science, King Saud University, Riyadh, Saudi Arabia; 5 IT4Innovations, VSB- Technical University of Ostrava, Ostrava, Czech Republic; 6 IS Department, College of Education, King Saud University, Riyadh, Saudi Arabia; 7 Department of Mathematics, College of Natural and Computational Sciences, Debre Berhan University, 445 Debre Berhan, Ethiopia; 8 Department of Computer Science and Mathematics, Lebanese American University, Byblos, Lebanon

**Keywords:** HIV infection, Mathematical delay model, Stochastic effect, Stability analysis, Spectral method, Legendre-Gauss-Lobatto points, Computational biology and bioinformatics, Mathematics and computing

## Abstract

The economic impact of Human Immunodeficiency Virus (HIV) goes beyond individual levels and it has a significant influence on communities and nations worldwide. Studying the transmission patterns in HIV dynamics is crucial for understanding the tracking behavior and informing policymakers about the possible control of this viral infection. Various approaches have been adopted to explore how the virus interacts with the immune system. Models involving differential equations with delays have become prevalent across various scientific and technical domains over the past few decades. In this study, we present a novel mathematical model comprising a system of delay differential equations to describe the dynamics of intramural HIV infection. The model characterizes three distinct cell sub-populations and the HIV virus. By incorporating time delay between the viral entry into target cells and the subsequent production of new virions, our model provides a comprehensive understanding of the infection process. Our study focuses on investigating the stability of two crucial equilibrium states the infection-free and endemic equilibriums. To analyze the infection-free equilibrium, we utilize the LaSalle invariance principle. Further, we prove that if reproduction is less than unity, the disease free equilibrium is locally and globally asymptotically stable. To ensure numerical accuracy and preservation of essential properties from the continuous mathematical model, we use a spectral scheme having a higher-order accuracy. This scheme effectively captures the underlying dynamics and enables efficient numerical simulations.

## Introduction

The global prevalence of HIV remains a critical public health challenge, necessitating continuous efforts to comprehend and control its dynamics. HIV poses a significant health threat to human around the globe with approximately 38 million people living with HIV worldwide as of 2022^1^. Despite substantial progress in understanding the virus and developing antiretroviral therapies, still serious challenges persist in mitigating the spread of HIV^2,3^. Mathematical modeling approach has a significant role in comprehending the complex dynamics of HIV transmission, helping researchers to explore various scenarios and interventions^4^. Modeling of real life problems using mathematical tools has proven to be a significant tool in understanding the intricate interactions within the HIV transmission dynamics, helping in the development of effective controlling strategies for prevention^5–8^.

Stochasticity accounts for the inherent randomness in the transmission dynamics of a disease whereas the time delays reflect the lag between infection and the manifestation of symptoms, along with the response time of control measures^9–12^. Moreover, the time delay is essential in capturing the temporal aspects of HIV transmission dynamics^13^. Time delays may arise due to a number of factors such as the latent period between infection and the onset of infectiousness, the time taken for a disease diagnosis, or delays in the implementation of preventive measures. Understanding the influence of time delays is essential for devising effective strategies to mitigate the incidence of the virus^14^.

The dynamics of infectious diseases, including HIV using novel modeling approaches have evolved over the years^15,16^. Classical compartmental models, such as the susceptible-infected-recovered (SIR) model, provide a foundation for studying disease spread. However, these models often oversimplify real-world complexities, prompting the development of more sophisticated models^16^. Stochastic modeling acknowledges the inherent randomness in disease transmission, incorporating probability distributions to account for uncertainties in the infection process^17–19^. This approach is particularly relevant for HIV, given the variability in individual behavior and contact patterns. Additionally, time delays can significantly impact the dynamics of infectious diseases, influencing the effectiveness of interventions and control measures^20–22^. In literature, different epidemic diseases were presented by the researchers such as the global dynamics of an epidemic age model was presented in^23^. The prediction and parameter estimation for COVID-19 pandemic in Algeria was addressed in^24–26^. The dynamics of a diffusive dispersal viral mathematical model was studied in^28^.

The primary objective of this research is to analyze the mathematical dynamics of a stochastic HIV model considering the effect of time delay. By incorporating a spectral collocation scheme, we aim to numerically solve the model equations and investigate the impact of these factors on disease dynamics. We develop a novel stochastic HIV model coupled with time delay. A comprehensive theoretical analysis is presented for the proposed model. The Analysis and transmission in the presence of of stochasticity and time delay on disease dynamics is shown graphically. Moreover, This research contributes to the existing body of knowledge by providing a detailed analysis of a mathematical model that considers these factors simultaneously. Building upon traditional deterministic models, this approach incorporates randomness in transmission events and disease progression timing, offering a more realistic framework for exploring HIV dynamics and evaluating intervention strategies. Through comprehensive simulations, we seek to elucidate the interplay between stochasticity, time delays, and intervention efficacy, with the ultimate goal of informing targeted public health interventions to mitigate the burden of HIV/AIDS globally.

The sections of this paper is structured as follows: “Stochastic HIV model with time delay” section presents the formulation of the stochastic HIV model with time delay, detailing the model assumptions and equations. “Qualitative analysis of the model” section introduces the qualitative analysis of the model. “Numerical treatment of the stochastic HIV delay model” section presents the spectral collocation scheme as the numerical solution for solving the model and depicts the results of numerical simulations, highlighting the impact of stochasticity and time delay on disease dynamics. Finally, “Conclusion” section concludes the paper, by summarizing key insights and suggesting avenues for future research. Through this comprehensive exploration, we aim to contribute to the scientific understanding of HIV dynamics and provide actionable insights for improving public health outcomes.

## Stochastic HIV model with time delay

This section briefly presents the mathematical model formulation to study viral dynamics within the host, incorporating the hypotheses, virus facts, and previous literature mathematical models^4,27^. However, despite an increasing number of studies in this field, there are still aspects that remain poorly understood. To facilitate the extraction of useful information and the testing of various hypotheses, mathematical models often include certain assumptions^29,30^. Observations suggest that in vitro, most of HIV-infected cells vanish before the virus production commences^31,32^. The virus-productive cells generate virions represented as *V* at rate $N\epsilon _I$ *N* represents the average count of infectious virions that are released by an infected cell throughout its lifespan. It is important to highlight that there is a general consensus among researchers that most of the virions generated by these infected cells do not possess the capability to cause infection^33,34^. Since these non-infectious virions do not contribute of new cells to the HIV infection, they are excluded from this mathematical model. The infectious virions *V* can either be removed from the virus-free cell population by the immune system at an inherent clearance denoted by *C*, where the infected target cells (CD4+T cells) are denoted by the parameter $\beta$. Here, target cell concentration is denoted by *T*, and $\Lambda$ is the new recrement rate that’s not yet infected, while $\mu _0$ denotes the mortality rate. In our constructed mathematical model, we categorize infected cells into two classes. The first class, denoted as $I_E$ is the proposed cells included in an eclipse phase that does not actively produce the proposed virus. Where the second class, labeled as *I* comprises cells that actively produce the virus. The eclipse phase transition of the cells to class *I*. On the other hand, cells in the *I* class die at a rate $\epsilon _I.$ Additionally, those cells which are in the eclipse stage can be eliminated by the immune system. It is crucial to note that the number of the target cells the virus does not infect *T* and depends on $\Lambda$ as well the specific death rate $\mu _0$ for target cells in the constructed mathematical model. Moreover, the addition of stochastic terms makes the model more realistic and shows the additional random properties.

The proposed a dynamics of HIV deterministic model^35^ is given by:

1 $$\begin{aligned} {\left\{ \begin{array}{ll} \frac{dI_{E}(t)}{dt}=2\beta _1 V(t)T(t)-\beta _2 V(t-\tau _0)T(t-\tau _0)e^{-\epsilon _{I_E}\tau _0}-\epsilon _{I_E}I_E(t),\\ \frac{dI(t)}{dt}=\beta _2 T(t-\tau _0)V(t-\tau _0)e^{-\epsilon _{I_E}\tau _0}-\epsilon _{I}I(t),\\ \frac{dV(t)}{dt}=N\epsilon _II(t)-CV(t)-\beta _1 V(t)T(t),\\ \frac{dT(t)}{dt}=\Lambda -\beta V(t)T(t)-\mu _0T(t). \end{array}\right. } \end{aligned}$$

Initial conditions are $I_E(0)>0, I(0)>0, V(0)>0, T(0)>0.$

In the context of this discussion, $\tau _0$ signifies the the eclipse phase duration. The term $e^{-\epsilon _{I_E}\tau _0}$ denotes the probability of an infected cell that could survive subsequently the viral entry for a time period $\tau _0$. It is essential to note that $\tau _0$ remains a constant time delay, resulting in a differential model with a $\tau _0$. For a more comprehensive grasp of the mathematical model at hand, we can gain insight by adding the stochastic term refer^36–39^.

Based on the above assumptions the HIV disease is subject to stochastic phenomena in a additive terms; the Eq. (1) is an be obtained a stochastic model given by:

2 $$\begin{aligned} {\left\{ \begin{array}{ll} \frac{dI_{E}(t)}{dt}=2\beta _1 T(t)V(t)-\beta _2 T(t-\tau _0)V(t-\tau _0)e^{-\epsilon _{I_E}\tau _0} -\epsilon _{I_E}I_E(t)+\upsilon I_E(t)\frac{dB(t)}{dt},\\ \frac{dI(t)}{dt}=\beta _2 T(t-\tau _0)V(t-\tau _0) e^{-\epsilon _{I_E}\tau _0}-\epsilon _{I}I(t)+\upsilon I(t)\frac{dB(t)}{dt},\\ \frac{dV(t)}{dt}=N\epsilon _II(t)-\beta _1 T(t)V(t)-CV(t)+\upsilon V(t)\frac{dB(t)}{dt},\\ \frac{dT(t)}{dt}=\Lambda -\beta V(t)T(t)-\mu _0T(t)+\upsilon T(t)\frac{dB(t)}{dt}. \end{array}\right. } \end{aligned}$$

In this study, we are focusing on higher-order spectral scheme for obtaining the solution to a stochastic HIV model described by Eq. (2). In this model, *B*(*t*) represents a Wiener process, which is a random process that exhibits erratic behavior over time. The Wiener process is represented by $\upsilon$ determining the degree of randomness or volatility in the problem. By developing an iterative solution to the model (2), we aim to better understand the dynamics of stochastic HIV system.

## Qualitative analysis of the model

This section covers some of the basic qualitative aspects of the HIV model which are crucial tool for investigating the behavior of a dynamical system. We deal with the stability of equilibrium points. These equilibrium solutions are associated with both deterministic and stochastic models, which are represented by equations labeled as Eqs. (1) and (2) respectively. By studying the stability results, we gain insights into how the system’s dynamics unfold over time, accounting for both deterministic and random cases.

The proposed model described by Eq. (1) can have up to two equilibrium solutions. The first one, called the infection-free equilibrium $E_0$, represents a steady state solution where there is no disease in the population. The second equilibrium solution is known as the endemic equilibrium, which occurs when the disease persists within the population.

### The basic reproduction number (${\mathscr {R}}_0$)

The system (1) denotes a simple HIV model. Now the infectious sub-system of Eq. (1) is

3 $$\begin{aligned} {\left\{ \begin{array}{ll} \frac{dI_{E}}{dt}=2\beta _1 TV-\beta _2 TVe^{-\epsilon _{I_E}\tau _0}-\epsilon _{I_E}I_E,\\ \frac{dI}{dt}=\beta _2 TVe^{-\epsilon _{I_E}\tau _0}-\epsilon _{I}I,\\ \frac{dV}{dt}=N\epsilon _II-CV-\beta _1 TV. \end{array}\right. } \end{aligned}$$

Eq. (3) has the Jacobian matrix:

4 $$\begin{aligned} J(I_E, I, V) = \begin{bmatrix} -\epsilon _{I_E} &{} 0 &{} 2\beta _1 T-\beta _2 Te^{-\epsilon _{I_E}\tau _0} \\ 0 &{} -\epsilon _I &{} \beta _2 Te^{-\epsilon _{I_E}\tau _0} \\ 0 &{} N\epsilon _I &{} -C-\beta _1 T \end{bmatrix}. \end{aligned}$$

The Jacobian matrix is further divided into two sub-matrices, the Transition matrix $\Gamma$ by:

$$\begin{aligned} \Gamma = \begin{bmatrix} 0 &{} 0 &{} 2\beta _1 T-\beta _2 Te^{-\epsilon _{I_E}\tau _0} \\ 0 &{} 0 &{} \beta _2 Te^{-\epsilon _{I_E}\tau _0} \\ 0 &{} 0 &{} -\beta _1 T \end{bmatrix}; \end{aligned}$$

and Transmission matrix $\beta _1$ by:

$$\begin{aligned} \Delta _1= \begin{bmatrix} -\epsilon _{I_E} &{} 0 &{} 0\\ 0 &{} -\epsilon _I &{} 0\\ 0 &{} N\epsilon _I &{} -C \end{bmatrix}. \end{aligned}$$

Transition matrix inversion is

$$\begin{aligned} \Delta _1^{-1}= \begin{bmatrix} \frac{-1}{\epsilon _{I_E}} &{} 0 &{} 0 \\ 0 &{} \frac{-1}{\epsilon _{I}} &{} 0 \\ 0 &{} \frac{-N}{C} &{} \frac{-1}{C} \end{bmatrix}. \end{aligned}$$

Now the NGM with large domain is denoted by $D_L$ is given by:

5 $$\begin{aligned}{} & {} D_L=-\Gamma \Delta _1^{-1}= \begin{bmatrix} 0 &{} \frac{\beta T(t)(2-e^{\epsilon _I\tau _0})N}{C} &{} \frac{\beta T(t)(2-e^{\epsilon _I\tau _0})}{C}\\ 0 &{} \frac{\beta T(t)e^{\epsilon _I\tau _0}N}{C} &{} \frac{\beta T(t)e^{\epsilon _I\tau _0}}{C} \\ 0 &{} -\frac{\beta T(t)N}{C} &{} -\frac{\beta T(t)}{C} \end{bmatrix}. \\{} & {} {\mathscr {R}}_0=trace (D_L)=\frac{\beta T(t)e^{\epsilon _I\tau _0}N}{C}-\frac{\beta T(t)}{C}. \end{aligned}$$

Following equation gives the reproduction number for the system given by Eq. (1)

6 $$\begin{aligned} {\mathscr {R}}_0=\frac{\beta \Lambda \big (e^{\epsilon _I\tau _0}N-1\big )}{\mu _0C}, \end{aligned}$$

where $$T(t)=\frac{\Lambda }{\mu _0}$$.

### Equilibrium points

The proposed model, (Eq. (1)), exhibits two distinct equilibriums states. The disease-free equilibrium, referred to as $E_0$, while second stationary state represents the endemic equilibrium, denoted as $E^*_1$. To identify these states, we need to find the critical values of model described by Eq. (1) by determining its stationary values.

#### **Theorem 1**

If ${\mathscr {R}}_0\le 1$, then equilibrium point $E_0$ is a stable solution for a system described by equations Eq. (1) on the entire region ${\mathbb {D}}$, which means that disease will not be spread and the population will remain healthy. On the other hand, if ${\mathscr {R}}_0>1$, then the endemic equilibrium solution $E^*_1$ (with values $I_E^*$, $I^*$, $V^*$, and $T^*$) of the model described by equations Eq. (1) is stable asymptotically on the region ${\mathbb {D}}$. This implies that the disease will persist in the population.

7 $$\begin{aligned}&I^*_E=\frac{\beta T^* V^*(2-e^{\epsilon _{I_E}\tau _0})}{\epsilon _{I_E}},\quad I^*=\frac{V^*(C+\beta T^*)}{N\epsilon _I},\quad V^*=\frac{\Lambda -\mu _0T^*}{\beta T^*},\quad R^*=\frac{\Lambda }{\beta V^*+\mu _0}. \end{aligned}$$

#### *Proof*

The HIV model Eq. (1), has stationary system is given by:

8 $$\begin{aligned}&2\beta V^*(t)T^*(t)-\beta V^*(t)T^*(t)e^{-\epsilon _{I_E}\tau _0}-\epsilon _{I_E}I^*_E(t)=0, \\&\beta V^*(t)T^*(t)e^{-\epsilon _{I_E}\tau _0}-\epsilon _{I}I^*(t)=0, \\&N\epsilon _II^*(t)-V^*(t)C-\beta V^*(t)T^*(t)=0, \\&\Lambda -\mu _0T^*(t)-\beta V^*(t)T^*(t)=0. \end{aligned}$$

To solve system in Eq. (8). We will discuss two major cases:

- infected classes $I^*$ and $I^*_E$ equal to zero
- $I^*_E$ and $I^*$ greater than zero.
  - If $I^*_E=0=I^*$: From the 2nd equation of system (8), we get $V^*=0$, where from last equation of Eq. (8), we get $T^*=\Lambda /\mu _0$. Therefore, we get the disease free equilibrium $E^*_0=(0, 0, 0, \Lambda /\mu _0,)$, having a case ${\mathscr {R}}_0<1.$
  - If $I_E^*>0$ and $I^*>0$, for the lake of calculation using Maple-13 software to found the proposed endemic equilibrium $E^*_1.$ In this case should be ${\mathscr {R}}_0>1$.

#### **Lemma 1**

Total region say ${\mathbb {D}}$ is positive invariance set for the proposed model given in Eq. (1).

#### *Proof*

For $N(t)=I_E(t)+I(t)+V(t)+T(t)$, then using model Eq. (1), we get:

9 $$\begin{aligned}{} & {} \frac{d}{dt}N(t)=\Lambda -\big (\epsilon _{I_E}I^*_E(t) +\epsilon _{I}I^*(t)+CV^*(t)+\mu _0T^*(t)\big )-N\epsilon _II^*(t). \end{aligned}$$

10 $$\begin{aligned}{} & {} \frac{d}{dt}N(t)\le \Lambda -\mu N(t), \end{aligned}$$

where $\mu =\min (\epsilon _{I_E}, \epsilon _{I}, C, \mu _0),$ therefore, Eq. (10) takes the form:

$$\begin{aligned} N(t)&\le \frac{\Lambda }{\mu }+N(0)e^{-\mu t}\le \frac{\Lambda }{\mu }. \end{aligned}$$

Hence in the total region ${\mathbb {D}}$ the system Eq. (1) is positively invariant.

The following lemma is proved by using method refer^42^.

#### **Lemma 2**

The system Eq. (1) solution $(I_E,I, V, T)$ has the aforementioned properties for $\big (I_E(0),I(0), V(0), T(0)\big )\in {\mathbb {R}}^4$:

$$\begin{aligned} \lim _{t\rightarrow \infty }\frac{1}{t}\int _{0}^{t} I_E(t)dB(t)=0, \end{aligned}$$

and similarly for each class.

#### **Definition 1**

The infected individuals in population $I_E$ and *I* are termed extinctive for model Eq. (2) iff $\lim _{t\rightarrow \infty }I_E(t) =0=\lim _{t\rightarrow \infty }I(t)=0$.

#### **Theorem 2**

Since, $\max \big (\frac{\beta \mu _0}{\Lambda }, \frac{\beta ^2}{2\mu ^2_0}\big )<\upsilon ^2$, or $\big (\frac{\beta \mu _0}{\Lambda }, \frac{\beta ^2}{2\mu ^2_0}\big )>\upsilon ^2$ with $\bar{{\mathscr {R}}_0}<1$, then both the infected classes $I_E$ and *I* of Eq. (2) are exponentially tends to zero. Conversely, if $\bar{{\mathscr {R}}_0}>1$, the each class of the model Eq. (2) are present, where the procedure of evaluating $\bar{{\mathscr {R}}_0}$ is presented in^19^ and is given by:

$$\begin{aligned} \bar{{\mathscr {R}}_0}={\mathscr {R}}_0-\frac{\upsilon ^2\Lambda ^2N-2\mu _0\beta \Lambda }{2\mu ^2_0C}. \end{aligned}$$

#### *Proof*

Suppose the solution of the proposed vaccination model Eq. (2) in the form of $\big \{I_E, I, V, T\big \}$ along with initial values $\big \{I_E(0), I(0), V(0), T(0)\big \}$. Utilizing the It$\hat{o}$ criteria we get:

11 $$\begin{aligned} d\ln I(t)&=\bigg (\beta Te^{\epsilon _{I_E}\tau _0}-\frac{C}{N}-\frac{\upsilon ^2T^2}{2}\bigg )dt+\upsilon TdB(t). \end{aligned}$$

Apply integral from 0 to *t*,

12 $$\begin{aligned} \ln I(t)&=\ln I(0)+\int _0^t\bigg (\beta Te^{\epsilon _{I_E}\tau _0}-\frac{C}{N}-\frac{\upsilon ^2T^2}{2}\bigg )dt+\int _0^t\upsilon TdB(t). \end{aligned}$$

Here we discuss the two cases, if $\upsilon ^2>\frac{\beta \mu _0}{\Lambda }$, then

13 $$\begin{aligned} \ln I(t)&\le \ln I(0)+\bigg (\frac{\beta ^2}{\upsilon ^2}(e^{\epsilon _{I_E}\tau _0}-0.5) -\frac{C}{N}\bigg )t+\int _{0}^{t}\upsilon TdB(t). \end{aligned}$$

Divide Eq. (13) by $t>0$, then

14 $$\begin{aligned} \frac{\ln I(t)}{t}&\le \frac{\ln I(0)}{t}+\bigg (\frac{\beta ^2}{\upsilon ^2}(e^{\epsilon _{I_E}\tau _0} -0.5)-\frac{C}{N}\bigg )+\frac{1}{t}\int _{0}^{t}\upsilon TdB(t). \end{aligned}$$

By taking $\lim _{t\rightarrow \infty }$ and using Lemma 2, then Eq. (14) converted to

$$\begin{aligned} \lim _{t\rightarrow \infty }{\frac{\ln I(t)}{t}}\le -\bigg (\frac{\mu _0C}{N}-\frac{\beta ^2}{\upsilon ^2}(e^{\epsilon _{I_E}\tau _0}-0.5)\bigg )<0. \end{aligned}$$

Which shows, $$\lim _{t\rightarrow \infty }I(t)=0.$$

In case 2, when $\upsilon ^2>\frac{\beta ^2}{2\mu ^2_0}$, and using Eq. (12) we get

15 $$\begin{aligned} \ln I(t)&\le \ln I(0)+\bigg (\frac{\beta \Lambda }{\mu _0}e^{\epsilon _{I_E}\tau _0} -\frac{\upsilon ^2\Lambda ^2}{2\mu ^2_0} -\frac{C}{N}\bigg )t+\int _{0}^{t}\upsilon TdB(t). \end{aligned}$$

Dividing Eq. (15) by $t>0$ we get

16 $$\begin{aligned} \frac{1}{t}\ln I(t)\le \frac{1}{t}\ln I(0)+\frac{C}{N}\bigg (\frac{\beta \Lambda }{\mu _0C}e^{\epsilon _{I_E}\tau _0}N -\frac{\upsilon ^2\Lambda ^2N}{2\mu ^2_0C} -1\bigg )+\frac{2}{t}\int _{0}^{t}\upsilon TdB(t). \end{aligned}$$

Applying $\lim _{t\rightarrow \infty }$ and then make use of Lemma 2, Eq. (16) gives

17 $$\begin{aligned}{l} \frac{1}{t}\ln I(t)\le \frac{C}{N}\bigg (\frac{\beta \Lambda }{\mu _{0}C}e^{\epsilon _{I_E}\tau _{0}}N -\frac{\upsilon ^{2}\Lambda ^{2}N}{2\mu ^{2}_{0}C} -1-\frac{\beta \Lambda }{\mu _{0}C}+\frac{\beta \Lambda }{\mu _{0}C}\bigg ). \\ \quad \frac{1}{t}\ln I(t)\le \frac{C}{N}\bigg (\frac{\beta \Lambda }{\mu _{0}C}(e^{\epsilon _{I_E}\tau _{0}}N-1) -\frac{\upsilon ^{2}\Lambda ^{2}N-2\mu _{0}\beta \Lambda }{2\mu ^{2}_{0}C}-1\bigg ). \\ \quad \lim _{t\rightarrow \infty }{\frac{\ln I(t)}{t}}\le \frac{C}{N}(\bar{{\mathscr {R}}_{0}}-1). \end{aligned}$$

Whenever, $$\bar{{\mathscr {R}}_0}<1$$, then

$$\begin{aligned} \lim _{t\rightarrow \infty }{\frac{1}{t}\ln I(t)}<0, \end{aligned}$$

which implies that $\lim _{t\rightarrow \infty }I(t)=0.$

Conversely, if $\bar{{\mathscr {R}}_0}<1$ then by using Eq. (17), we we get;

18 $$\begin{aligned} \lim _{t\rightarrow \infty }{\frac{\ln I(t)}{t}}\le \frac{C}{N}K_1, \end{aligned}$$

where, $K_1$ is any positive constant. From Eq. (18), we clearly observe that the infected class exist and positive non-zero in the population. This complete the proof.

## Numerical treatment of the stochastic HIV delay model

This section provides the numerical scheme of the stochastic HIV delay model (2) using a high order spectral collocation approach. Moreover, this section also present the visual dynamics of the model to validate the theoretical results.

### Numerical scheme using spectral method

Before apply spectral method, to provide an preview of Legendre polynomials given in^40,41^. The $n{th}$ order Legendre polynomials denoted by $P_{n}({\tau _a})$. Where the function $u(\tau _a)$ is approximated by:

19 $$\begin{aligned} u(\tau _a)=\sum _{i=0}^{n}u_i P_i(\tau _a). \end{aligned}$$

$u_{i}$ indicates Legendre coefficients, $\tau _{ai}, i=0,..., n$ are collocation nodes and $P_{n}(\tau _a)$ denotes $n{th}$-order. Where the Legendre polynomials are:

20 $$\begin{aligned}{} & {} P_{i}(\tau _a)=\frac{1}{2^{i}}\sum _{a=0}^{[\frac{i}{2}]}(-1)^{a}(_{a}^{i})(_{\quad i}^{2(i-a)})\tau ^{i-2a}, \quad \big (i=0, 1,..., n\big ), \quad \tau _a\in \big [-1,1\big ]. \\{} & {} \frac{i}{2}= {\left\{ \begin{array}{ll} \frac{i}{2}, \qquad \quad i\quad { Even},\\ \frac{i-1}{2}, \qquad \quad i\quad { Odd}. \end{array}\right. } \end{aligned}$$

Spectral method procedure we considered the Legendre-Gauss-Lobatto points $\{t_j\}_{j=0}^{N}$.

For this, taking integral on Eq. (2) from [0, *t*].

21 $$\begin{aligned} {\left\{ \begin{array}{ll} I_E(t)&{}=I_E(0)+\int _{0}^{t}\big (2\beta T(s)V(s)-\beta T(s-\tau _0)V(s-\tau _0)e^{-\epsilon _{I_E}\tau _0} -\epsilon _{I_E}I_E(s)\big ){ds}\\ &{}+\int _{0}^{t}\upsilon I_E(s)dB(s),\\ I(t)&{}=I(0)+\int _{0}^{t}\big (\beta T(s-\tau _0)V(s-\tau _0) e^{-\epsilon _{I_E}\tau _0}-\epsilon _{I}I(s)\big ){ds}+\int _{0}^{t}\upsilon I(s)dB(s),\\ V(t)&{}=V(0)+\int _{0}^{t}\big (N\epsilon _II(s)-CV(s) -\beta T(s)V(s)\big ){ds}+\int _{0}^{t}\upsilon V(s)dB(s),\\ T(t)&{}=T(0)+\int _{0}^{t}\big (\Lambda -\beta V(s)T(s)\big ){ds}-\mu _0T(s)+\int _{0}^{t}\upsilon T(s) dB(s), \end{array}\right\}} \end{aligned}$$

where $I_E(0), I(0), V(0), T(0)$ are the initial conditions subject to each class respectively. To convert the present interval to $[-1, 1]$ interval, we transform *s* like: $s=\frac{t}{2}(1+\varpi )$, then Eq. (21) takes the form:

22 $$\begin{aligned} I_E(t)&=I_E(0)+\frac{1}{2}t\int _{-1}^{1}\bigg (2\beta T\big (\frac{t}{2}(1+\varpi )\big )V\big (\frac{t}{2}(1+\varpi )\big )-\beta T\big (\frac{t}{2}(1+\varpi )-\tau _0\big ) \\ & \quad \times V\big (\frac{t}{2}(1+\varpi )-\tau _0\big )e^{-\epsilon _{I_E}\tau _0} -\epsilon _{I_E}I_E\big (\frac{t}{2}(1+\varpi )\big )\bigg )d\varpi +\frac{1}{2}t\int _{-1}^{1}\upsilon I_E\bigg (\frac{t(1+\varpi )}{2}\bigg )dB(\varpi ), \\ I(t)&=I(0)+\frac{1}{2}t\int _{-1}^{1}\bigg (\beta T\big (\frac{t}{2}(1+\varpi )-\tau _0\big )V\big (\frac{t}{2}(1+\varpi )-\tau _0\big ) e^{-\epsilon _{I_E}\tau _0}-\epsilon _{I}I\big (\frac{t}{2}(1+\varpi )\big )\bigg ){d\varpi } \\ & \quad +\frac{1}{2}t\int _{-1}^{1}\upsilon I\bigg (\frac{t(1+\varpi )}{2}\bigg )dB(\varpi ), \\ V(t)&=V(0)+\frac{1}{2}t\int _{-1}^{1}\bigg (N\epsilon _II\big (\frac{t}{2}(1+\varpi )\big ) -CV\big (\frac{t}{2}(1+\varpi )\big )-\beta T\big (\frac{t}{2}(1+\varpi )\big )V\big (\frac{t}{2}(1+\varpi )\big )\bigg ){d\varpi } \\& \quad +\frac{1}{2}t\int _{-1}^{1}\upsilon V\bigg (\frac{t(1+\varpi )}{2}\bigg )dB(\varpi ), \\ T(t)&=T(0)+\frac{1}{2}t\int _{-1}^{1}\bigg (\Lambda -\beta T\big (\frac{t}{2}(1+\varpi )\big )V\big (\frac{t}{2}(1+\varpi )\big ) -\mu _0T\big (\frac{t}{2}(1+\varpi )\big )\bigg ){d\varpi } \\ & \quad +\frac{1}{2}t\int _{-1}^{1}\upsilon T\bigg (\frac{t(1+\varpi )}{2}\bigg )dB(\varpi ), \end{aligned}$$

where the semi discretized spectral system Eq. (22) are:

23 $$\begin{aligned} I_E(t)&=I_E(0)+\frac{1}{2}t\sum _{k=0}^{N}\bigg (2\beta T\big (\frac{t}{2}(1+\varpi )\big )V\big (\frac{t}{2}(1+\varpi )\big )-\beta T\big (\frac{t}{2}(1+\varpi )-\tau _0\big ) \\ & \quad \times V\big (\frac{t}{2}(1+\varpi )-\tau _0\big )e^{-\epsilon _{I_E} \tau _0}-\epsilon _{I_E}I_E\big (\frac{t}{2}(1+\varpi )\big )\bigg )\omega _k +\frac{1}{2}t\sum _{k=0}^{N}\upsilon I_E\bigg (\frac{t(1+\varpi )}{2}\bigg )\omega ^*_k, \\ I(t)&=I(0)+\frac{1}{2}t\sum _{k=0}^{N}\bigg (\beta T\big (\frac{t}{2}(1+\varpi )-\tau _0\big )V\big (\frac{t}{2}(1+\varpi )-\tau _0\big ) e^{-\epsilon _{I_E}\tau _0}-\epsilon _{I}I\big (\frac{t}{2}(1+\varpi )\big )\bigg )\omega _k \\& \quad +\frac{1}{2}t\sum _{k=0}^{N}\upsilon I\bigg (\frac{t(1+\varpi )}{2}\bigg )\omega ^*_k, \\ V(t)&=V(0)+\frac{1}{2}t\sum _{k=0}^{N}\bigg (N\epsilon _II\big (\frac{t}{2}(1+\varpi )\big ) -CV\big (\frac{t}{2}(1+\varpi )\big )-\beta T\big (\frac{t}{2} (1+\varpi )\big )V\big (\frac{t}{2}(1+\varpi )\big )\bigg )\omega _k \\ & \quad +\frac{1}{2}t\sum _{k=0}^{N}\upsilon V\bigg (\frac{t(1+\varpi )}{2}\bigg )\omega ^*_k, \\ T(t)&=T(0)+\frac{1}{2}t\sum _{k=0}^{N}\bigg (\Lambda -\beta T\big (\frac{t}{2}(1+\varpi )\big )V\big (\frac{t}{2}(1+\varpi )\big ) -\mu _0T\big (\frac{t}{2}(1+\varpi )\big )\bigg )\omega _k \\ & \quad +\frac{1}{2}t\sum _{k=0}^{N}\upsilon T\bigg (\frac{t(1+\varpi )}{2}\bigg )\omega ^*_k. \end{aligned}$$

The weight function refer^15,16^, for Eq. (1) is given by

$$\begin{aligned} \omega _{k}=\frac{2}{[L^{'}_{1+N}(s_{k})]^{2}(1-s^{2}_{k})},\quad \quad k \in [0, N]. \end{aligned}$$

*L* is the Lagrange polynomials.

Also the weight function for Eq. (2) is

$$\begin{aligned} \omega ^*_k = \sqrt{\omega _{k}}\times randn(1,N). \end{aligned}$$

The spectral solution of each $S, E, I, I_{A}, V$ and *R* by using the above Eq. (21)

24 $$\begin{aligned} I_E=\sum _{n=0}^{N}(I_E)_nP_n(t),\quad I=\sum _{n=0}^{N}I_nP_n(t),\quad V=\sum _{n=0}^{N}V_nP_n(t),\quad T=\sum _{n=0}^{N}T_nP_n(t). \end{aligned}$$

The respective Legendre polynomial coefficients of each of functions $I_E$, *I*, *V* and *T* are in the form: $I_E$, *I*, *V*, *T*, respectively. Now using the solution Eq. (24) then:

25 $$\begin{aligned} \sum _{n=0}^{N}(I_E)_nP_n(t)&=\sum _{n=0}^{N}(I_E)_nP_n(0)+\frac{1}{2}t \sum _{k=0}^{N}\bigg (2\beta \sum _{n=0}^{N}T_nP_n(\zeta _k)\sum _{n=0}^{N}V_nP_n(\zeta _k) \\ & \quad -\beta \sum _{n=0}^{N}I_nP_n(\zeta _k-\tau _0)\sum _{n=0}^{N}V_nP_n(\zeta _k-\tau _0)e^{-\epsilon _{I_E} \tau _0}-\epsilon _{I_E}\sum _{n=0}^{N}(I_E)_nP_n(\zeta _k)\bigg )\omega _k \\& \quad +\frac{1}{2}t\sum _{k=0}^{N}\upsilon \sum _{n=0}^{N}(I_E)_nP_n(\zeta _k)\omega ^*_k, \\ \sum _{n=0}^{N}I_nP_n(t)&=\sum _{n=0}^{N}I_nP_n(0)+\frac{1}{2}t\sum _{k=0}^{N}\bigg (\beta \sum _{n=0}^{N}T_nP_n(\zeta _k-\tau _0)\sum _{n=0}^{N}V_nP_n(\zeta _k-\tau _0) e^{-\epsilon _{I_E}\tau _0} \\& \quad -\epsilon _{I}\sum _{n=0}^{N}I_nP_n(\zeta _k)\bigg )\omega _k+\frac{1}{2} t\sum _{k=0}^{N}\upsilon \sum _{n=0}^{N}I_nP_n(\zeta _k)\omega ^*_k, \\ \sum _{n=0}^{N}V_nP_n(t)&=\sum _{n=0}^{N}V_nP_n(0)+\frac{1}{2} t\sum _{k=0}^{N}\bigg (N\epsilon _I\sum _{n=0}^{N}I_nP_n(\zeta _k) -C\sum _{n=0}^{N}V_nP_n(\zeta _k) \\& \quad -\beta \sum _{n=0}^{N}T_nP_n(\zeta _k)\sum _{n=0}^{N}V_nP_n(\zeta _k)\bigg ) \omega _k+\frac{1}{2}t\sum _{k=0}^{N}\upsilon \sum _{n=0}^{N}V_nP_n(\zeta _k)\omega ^*_k, \\ \sum _{n=0}^{N}T_nP_n(t)&=\sum _{n=0}^{N}T_nP_n(0)+\frac{1}{2}t\sum _{k=0}^{N}\bigg (\Lambda -\beta \sum _{n=0}^{N}T_nP_n(\zeta _k)\sum _{n=0}^{N}V_nP_n(\zeta _k) \\& \quad -\mu _0\sum _{n=0}^{N}T_nP_n(\zeta _k)\bigg )\omega _k+\frac{1}{2}t\sum _{k=0}^{N}\upsilon \sum _{n=0}^{N}T_nP_n(\zeta _k)\omega ^*_k. \end{aligned}$$

We take $\zeta _k=\big (\frac{t(1+\varpi )}{2}\big )$. The proposed system Eq. (25) gives of $4N+ 4$ of unknowns in which 4*N* nonlinear equations. Using initial conditions say:

26 $$\begin{aligned} \sum _{n=0}^{N}(I_E)_nP_n(0)=\lambda _1.\quad \sum _{n=0}^{N}(I)_nP_n(0)=\lambda _2, \quad \sum _{n=0}^{N}V_nP_n(0)=\lambda _3, \quad \sum _{n=0}^{N}T_nP_n(0)=\lambda _4. \end{aligned}$$

Equations (25) and (26) forms a $(4N+4)$ equations. Therefore, solving the above two systems gives the solution of corresponding all unknowns. In last using the above unknown values in Eq. (24), and get a solution to the model given in Eq. (2).

### Numerical results

**Figure 1:**
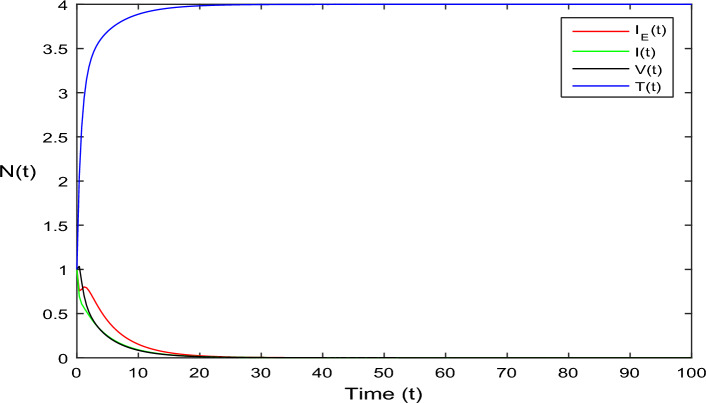
Dynamics of deterministic HIV model (1) that is ${\mathscr {R}}_0<1$.

**Figure 2:**
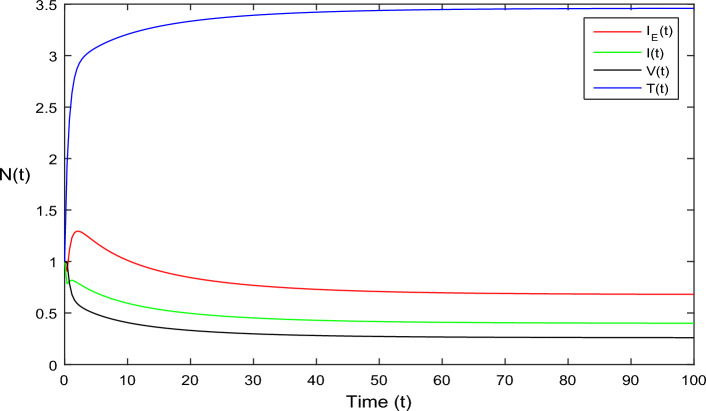
Dynamics of deterministic HIV model (1) that is ${\mathscr {R}}_0>1.$

**Figure 3:**
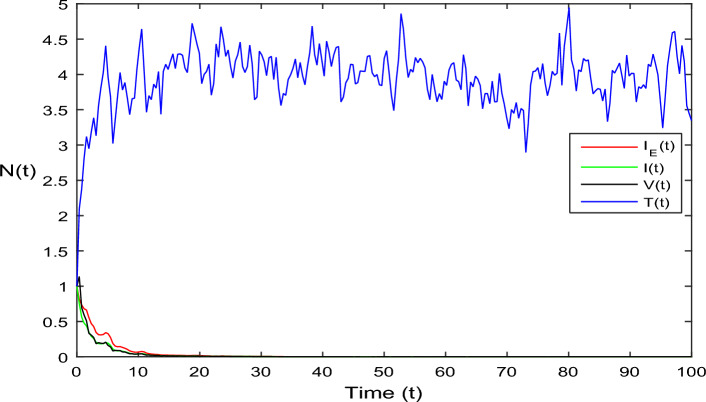
Dynamics of HIV system Eq. (2) in stochastic case where $\bar{{\mathscr {R}}}_0<1.$

**Figure 4:**
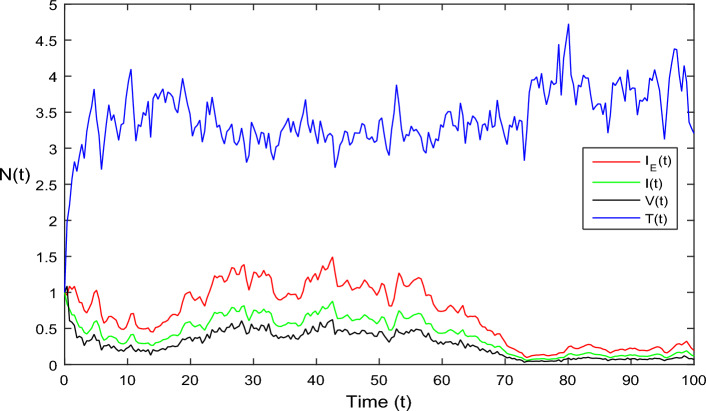
Simulation of HIV model Eq. (2) in stochastic case where $\bar{{\mathscr {R}}}_0>1.$

**Figure 5:**
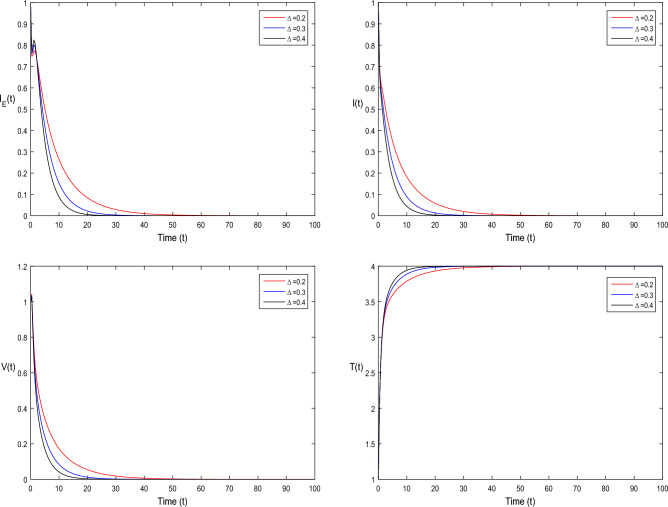
Simulation of deterministic HIV system (1) for different values of $\tau _0$, where ${\mathscr {R}}_0<1$.

**Figure 6:**
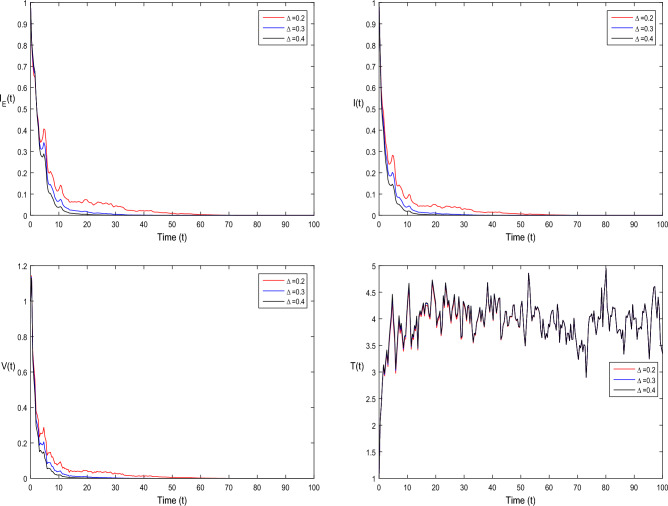
Dynamics of stochastic HIV system (2) for different values of $\tau _0$, where ${\mathscr {R}}_0<1$.

**Figure 7:**
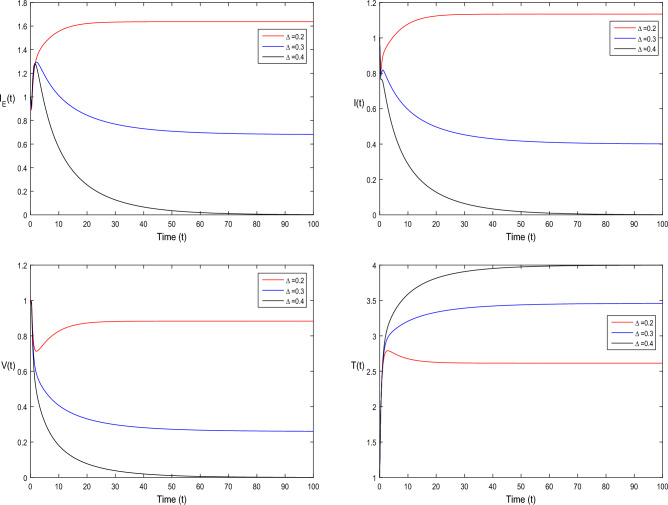
Dynamics of deterministic HIV system (1) for different values of $\tau _0$, where ${\mathscr {R}}_0>1$.

**Figure 8:**
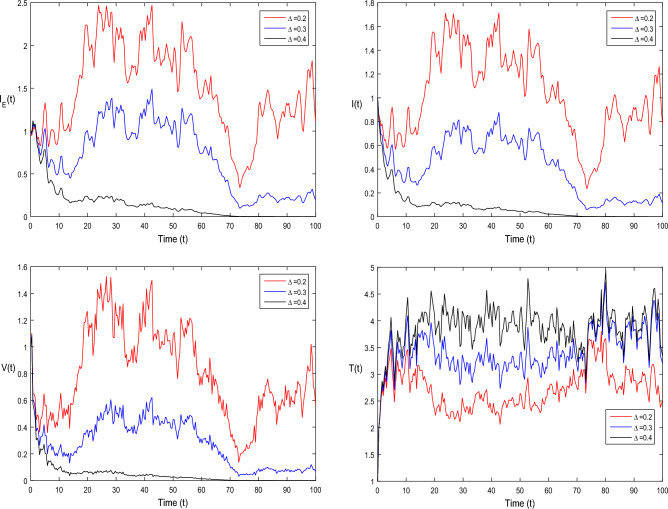
Simulation of stochastic HIV system (2) for different values of $\tau _0$, where ${\mathscr {R}}_0>1$.

The current section focuses on presenting some numerical problems and their corresponding graphical results. The numerical results are captured and explained for both deterministic systems Eq. (1) and the stochastic system Eq. (2). To find these numerical results, the spectral collocation method is employed. The obtained results are then visualized in Figs. 1, 2, 3, 4, 5, 6, 7, 8. The computations for this study are performed on a personal computer using Maple and Matlab software. To simplify the calculations, each initial value is assumed to be equal to 1. The tables contain the parameter values

**Figure 9:**
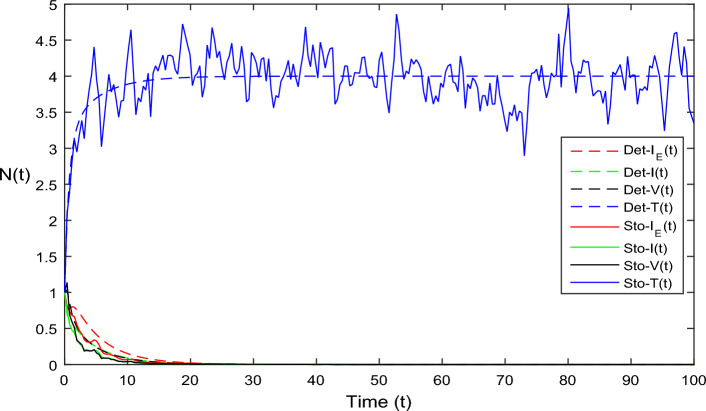
Solution comparison of both HIV systems given in (1) and (2), for ${\mathscr {R}}_0<1.$

**Figure 10:**
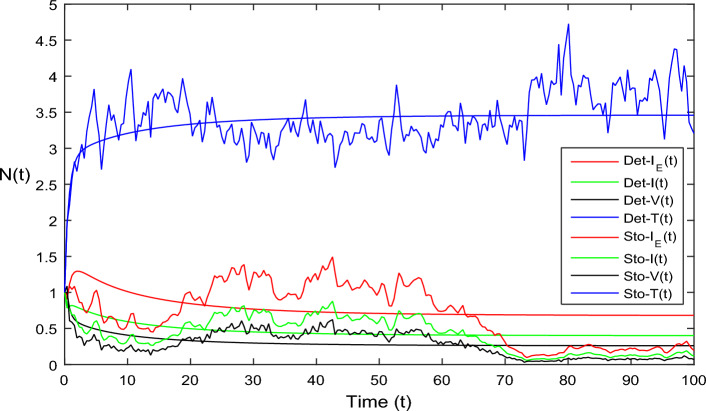
Solution comparison of both HIV systems given in (1) and (2), for ${\mathscr {R}}_0>1.$

In Fig. 1, the parameter values in Table 1 are assumed for deterministic system Eq. (1). The reproduction number becomes ${\mathscr {R}}_0<1$, by using given parameter values. Further, by using Theorem 4.1, we see the model Eq. (1) has a stable infectious-free equilibrium point $E_0(0, 0, 0, 4)$ where $I_E(t)$ is in the form $\Lambda /\mu _0=4$. For Fig. 2, we assume the parameter values given in Table 2, we got ${\mathscr {R}}_0>1$, and using theorem 4.1 the model Eq. (1) has a stable endemic equilibrium point as all the compartments are tends to zero as shown in Fig. 2. For Fig. 3, using the Table 1, parameter values for stochastic system Eq. (2). The above simulation shows that $\max \big (\frac{\beta \mu _0}{\Lambda }, \frac{\beta ^2}{2\mu ^2_0}\big )<\upsilon ^2$ and $\bar{{\mathscr {R}}_0}<1$ along with theorem 4.5 the infected classes of model Eq. (2) become zero. Likewise, for Fig. 4, employing the parameter values outlined in Table 2, the stochastic model Eq. (2) adhere to $\left( \frac{\beta \mu _0}{\Lambda }, \frac{\beta ^2}{2\mu ^2_0}\right) > \upsilon ^2$, satisfying $\bar{{\mathscr {R}}_0} > 1$. Utilizing theorem 4.5, this demonstrates that each class converge to endemic equilibrium as described in equations Eq. (2), which is illustrated in Fig. 4. In Fig. 5, we draw the graphs for different values of delay parameter $\tau _0= 0.2, 0.3, 0.4$, using the parameter values of Table 1, for the system Eq. (1). We clearly see that, if we increase the value of $\tau _0$, then $I_E(t), I(t), V(t)$ classes becomes decreases, correspondingly *T*(*t*) class become increase. Such dissertation is clearly seen in Fig. 5. Similarly, in Fig. 6 using parameter values given in Table 1, for the stochastic system Eq. (2). Again we see that if we increase the value of $\tau _0$, then $I_E(t), I(t), V(t)$ classes are decreasing, correspondingly *T*(*t*) becomes increase. In Fig. 7, we draw the graphs for different values of delay parameter $\tau _0= 0.2, 0.3, 0.4$, using the parameter values of Table 2, for the deterministic system Eq. (1). We clearly see that, if we increase the value of $\tau _0$, then $I_E(t), I(t), V(t)$ classes become decreases, correspondingly *T*(*t*) class become increase. Such dissertation is clearly seen in Fig. 7. For the above parameter values the proposed system satisfies the endemic equilibrium $E_1^*$. Similarly, for Fig. 8, and Table 2, values are assumed for the stochastic system Eq. (2). Again we see that if we increase the value of $\tau _0$, then $I_E(t), I(t), V(t)$ classes are decreasing, consequently *T*(*t*) becomes increase. Figure 9 is drawn for the parameter values given in Table 1. For this figure, we show the comparison of the solutions Eqs. (1) and (2). The simple computation shows that for model Eq. (1) ${\mathscr {R}}_0<1$, where for stochastic model Eq. (2) $\max \big (\frac{\beta \mu _0}{\Lambda }, \frac{\beta ^2}{2\mu ^2_0}\big )<\upsilon ^2$ with $\bar{{\mathscr {R}}_0}<1.$ Using the theorems 4.1 and 4.5, we see both the systems satisfy the disease-free equilibriums. Figure 10 is drawn for the Table 2 parameter values. The present figure shows the comparison of the solutions Eqs. (1) and (2). The simple calculations described that the model Eq. (1) have ${\mathscr {R}}_0>1$, where for stochastic model Eq. (2) $\max \big (\frac{\beta \mu _0}{\Lambda }, \frac{\beta ^2}{2\mu ^2_0}\big )>\upsilon ^2$ with $\bar{{\mathscr {R}}_0}>1.$ Again using the Theorems 4.1 and 4.5, we see both the systems satisfy the endemic equilibriums.

**Table 1:** Parameter values for ${\mathscr {R}}_0<1$.

Parameters	Values
$\Lambda$	4
$\beta$	0.3
$\mu _0$	1
$\tau _0$	0.3
$\epsilon _I$	0.8
$\epsilon _{I_E}$	0.9
C	1
N	2
$\upsilon$	1

**Table 2:** Parameter values for ${\mathscr {R}}_0>1$.

Parameters	Values
$\Lambda$	4
$\beta$	0.7
$\mu _0$	1
$\tau _0$	0.3
$\epsilon _I$	0.9
$\epsilon _{I_E}$	0.8
C	1
N	2
$\upsilon$	1

## Conclusion

In conclusion, our research provides a nuanced understanding of the mathematical dynamics of stochastic HIV transmission, considering both time delay and stochasticity. The stability analysis, supported by theorems and lemmas, yields valuable insights into the potential success of control strategies. The findings emphasize the critical importance of timely interventions and sustained efforts in the ongoing global fight against HIV.

We can confidently state that the disease-free equilibrium is proven to be stable asymptotically when ${\mathscr {R}}_0$ becomes less than 1. Conversely, when ${\mathscr {R}}_0$ exceeds 1, the stable endemic equilibrium is established. These results provide significant insights in understanding the dynamics of HIV infection and its potential spread in different population settings. Numerical simulations were performed to examine the influence of the delay parameter ($\tau _0$) on the proposed models. Various values of $\tau _0$ were tested, and the impact of this delay was investigated.

As we conclude this research, we call for continued interdisciplinary collaboration between mathematicians, epidemiologists, and public health professionals. The synergy of theoretical insights and empirical data is essential for refining models, validating assumptions, and ultimately informing evidence-based interventions. Our journey in understanding the mathematical intricacies of HIV transmission continues, fueled by the collective commitment to creating a world free from the burden of HIV/AIDS.

In future work, we aim to extend the proposed stochastic HIV model with time delay to corporate the additional complexities such as varying levels of intervention coverage and spatial heterogeneity. Such expansions will allow for a more informative understanding about HIV transmission dynamics across different population subgroups and geographic regions. Furthermore, we extend to integrate real-world data sources to calibrate the model and validate, to enhancing its predictive capabilities for informing evidence based public health strategies.

## Data Availability

The data that support the findings of this study are available from the corresponding author upon reasonable request. Further, no experiments on humans and/or the use of human tissue samples involved in this study.
